# COVID-19 Outbreak Associated with Air Conditioning in Restaurant, Guangzhou, China, 2020

**DOI:** 10.3201/eid2607.200764

**Published:** 2020-07

**Authors:** Jianyun Lu, Jieni Gu, Kuibiao Li, Conghui Xu, Wenzhe Su, Zhisheng Lai, Deqian Zhou, Chao Yu, Bin Xu, Zhicong Yang

**Affiliations:** Guangzhou Center for Disease Control and Prevention, Guangzhou, China (J. Lu, K. Li, C. Xu, W. Su, C. Yu, Z. Yang);; Guangzhou Yuexiu District Center for Disease Control and Prevention, Guangzhou, China (J. Gu, Z. Lai, D. Zhou, B. Xu)

**Keywords:** family cluster, outbreak, restaurant, droplet transmission, 2019 novel coronavirus disease, COVID-19, severe acute respiratory syndrome coronavirus 2, SARS-CoV-2, viruses, respiratory infections, zoonoses

## Abstract

During January 26–February 10, 2020, an outbreak of 2019 novel coronavirus disease in an air-conditioned restaurant in Guangzhou, China, involved 3 family clusters. The airflow direction was consistent with droplet transmission. To prevent the spread of the virus in restaurants, we recommend increasing the distance between tables and improving ventilation.

From January 26 through February 10, 2020, an outbreak of 2019 novel coronavirus disease (COVD-19) affected 10 persons from 3 families (families A–C) who had eaten at the same air-conditioned restaurant in Guangzhou, China. One of the families had just traveled from Wuhan, Hubei Province, China. We performed a detailed investigation that linked these 10 cases together. Our study was approved by the Ethics Committee of the Guangzhou Center for Disease Control and Prevention.

On January 23, 2020, family A traveled from Wuhan and arrived in Guangzhou. On January 24, the index case-patient (patient A1) ate lunch with 3 other family members (A2–A4) at restaurant X. Two other families, B and C, sat at neighboring tables at the same restaurant. Later that day, patient A1 experienced onset of fever and cough and went to the hospital. By February 5, a total of 9 others (4 members of family A, 3 members of family B, and 2 members of family C) had become ill with COVID-19.

The only known source of exposure for the affected persons in families B and C was patient A1 at the restaurant. We determined that virus had been transmitted to >1 member of family B and >1 member of family C at the restaurant and that further infections in families B and C resulted from within-family transmission. 

Restaurant X is an air-conditioned, 5-floor building without windows. The third floor dining area occupies 145 m^2^; each floor has its own air conditioner (Figure). The distance between each table is about 1 m. Families A and B were each seated for an overlapping period of 53 minutes and families A and C for an overlapping period of 73 minutes. The air outlet and the return air inlet for the central air conditioner were located above table C (Figure, panel B). 

**Figure:**
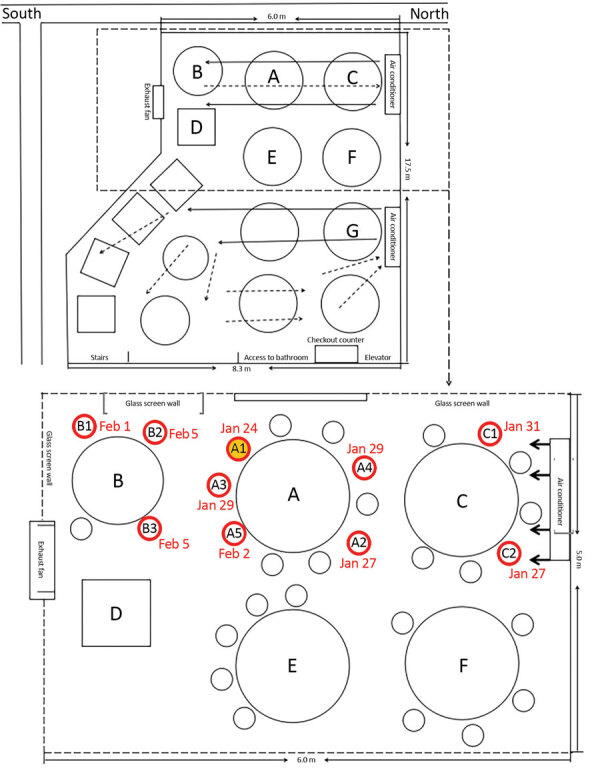
Sketch showing arrangement of restaurant tables and air conditioning airflow at site of outbreak of 2019 novel coronavirus disease, Guangzhou, China, 2020. Red circles indicate seating of future case-patients; yellow-filled red circle indicates index case-patient.

On January 24, a total of 91 persons (83 customers, 8 staff members) were in the restaurant. Of these, a total of 83 had eaten lunch at 15 tables on the third floor. Among the 83 customers, 10 became ill with COVID-19; the other 73 were identified as close contacts and quarantined for 14 days. During that period, no symptoms developed, and throat swab samples from the contacts and 6 smear samples from the air conditioner (3 from the air outlet and 3 from the air inlet) were negative for severe acute respiratory syndrome coronavirus 2 by reverse transcription PCR.

From our examination of the potential routes of transmission, we concluded that the most likely cause of this outbreak was droplet transmission. Although the index patient (patient A1) was asymptomatic during the lunch, presymptomatic transmission has been reported (*1*). Given the incubation periods for family B (Appendix Figure**)**, the most likely scenario is that all 3 family B members were directly infected by patient A1. However, we cannot not exclude the possibility that patients B2 and B3 were infected by patient B1, the first family B member to become ill. For family C, a possible scenario is that both patients C1 and C2 were infected by patient A1; another scenario is that the patient C1 acquired the infection while caring for patient C2, beginning on January 27.

Virus transmission in this outbreak cannot be explained by droplet transmission alone. Larger respiratory droplets (>5 μm) remain in the air for only a short time and travel only short distances, generally <1 m (*2**,**3*). The distances between patient A1 and persons at other tables, especially those at table C, were all >1 m. However, strong airflow from the air conditioner could have propagated droplets from table C to table A, then to table B, and then back to table C (Figure).

Virus-laden small (<5 μm) aerosolized droplets can remain in the air and travel long distances, >1 m (*4*). Potential aerosol transmission of severe acute respiratory syndrome and Middle East respiratory syndrome viruses has been reported (*5**,**6*). However, none of the staff or other diners in restaurant X were infected. Moreover, the smear samples from the air conditioner were all nucleotide negative. This finding is less consistent with aerosol transmission. However, aerosols would tend to follow the airflow, and the lower concentrations of aerosols at greater distances might have been insufficient to cause infection in other parts of the restaurant.

Our study has limitations. We did not conduct an experimental study simulating the airborne transmission route. We also did not perform serologic studies of swab sample–negative asymptomatic family members and other diners to estimate risk for infection. 

We conclude that in this outbreak, droplet transmission was prompted by air-conditioned ventilation. The key factor for infection was the direction of the airflow. Of note, patient B3 was afebrile and 1% of the patients in this outbreak were asymptomatic, providing a potential source of outbreaks among the public (*7**,**8*). To prevent spread of COVID-19 in restaurants, we recommend strengthening temperature-monitoring surveillance, increasing the distance between tables, and improving ventilation. 

Appendix Timeline of outbreak of 2019 novel coronavirus disease associated with air conditioning in restaurant and clinical and laboratory results for patients, Guangzhou, China, 2020.
