# Supplementary material for: COVID-19 Outbreak Associated with Air Conditioning in Restaurant, Guangzhou, China, 2020
Source: Emerg Infect Dis. 2020 Jul;26(7):1628–31. doi: 10.3201/eid2607.200764 (PMC7323555; doi:10.3201/eid2607.200764)
Supplement: Appendix — Timeline of outbreak of 2019 novel coronavirus disease associated with air conditioning in restaurant and clinical and laboratory results for patients, Guangzhou, China, 2020. [file 20-0764-Techapp-s1.pdf]

Article DOI: https://doi.org/10.3201/eid2607.200764

COVID-19 Outbreak Associated with Air Conditioning in Restaurant, Guangzhou, China, 2020

Appendix

Appendix Table. Summary of clinical features and laboratory results of the three family clusters infected with COVID-19 at presentation

Variable	Family A					Family B			Family C	
Relationship	Patient A1 (Index case)*	Patient A2	Patient A3	Patient A4	Patient A5	Patient B1	Patient B2	Patient B3	Patient C1	Patient C2
	Sister of patient A2	Sister of patient A1	Wife of patient A5, in-laws of patient A1	Niece of patient A1	Husband of patient A3, in-laws of patient A1	Wife of patient B3	Daughter of patient B1 and B3	Husband of patient B1	Daughter of patient C2	Mother of patient C1
Age (years)	63	60	62	34	63	44	20	53	54	82
Sex	Female	Female	Female	Female	Male	Female	Female	Male	Female	Female
Occupation	Retiree	Retiree	Retiree	Staff	Retiree	Cook	Student	self-em-ployed house-hold	Civil servant	Retiree
Chronic medical illness	Hyper-tension, hyper-lipidemia	None	None	None	Hypertension	None	None	None	None	None
interval between admission to hospital and symptom onset (days)	1	0	0	0	0	3	3	1	1	9
Presenting symptoms and signs										
Time of onset	Jan. 24	Jan. 27	Jan. 29	Jan. 29	Feb. 2	Feb. 1	Feb. 3	Feb. 5	Jan. 31	Jan. 27
Fever	+	+	+	+	+	+	+	-	+	+
Cough	+	-	-	-	-	+	+	-	+	-
Running nose	-	-	-	-	-	-	-	-	-	+
Polypnea	-	-	-	-	-	-	+	+	-	-
Head pain	-	-	-	-	-	-	-	+	-	-
Chest pain	-	-	-	-	-	-	-	+	-	-
Diarrhea	-	-	-	-	-	-	+	-	-	-

Variable	Family A					Family B			Family C	
Body temperature (°C)	37.80	37.80	37.30	37.70	37.80	38.40	38.60	-	39.90	37.8
leukocyte count ($\times 10^9$ cells per L)	4.08	4.79	4.76	5.19	6.51	3.52	6.39	6.53	5.40	4.7
Lymphocyte count ($\times 10^9$ cells per L)	0.93	1.05	1.77	1.28	2,74	1.13	1.36	1.96	1.49	0.7
Lymphocyte%	33.10	21.90	37.20	24.70	42.10	32.10	21.30	3.88	27.60	15.50
Neutrophil count ($\times 10^9$ cells per L)	1.60	3.34	2.58	2.92	2.80	2.09	4.02	30.00	3.64	3.6
Neutrophil %	56.9	69.80	54.40	56.20	43.00	59.40	62.90	59.40	67.40	74.90

*Detail of the index case: the index case was the member of family A, who came from Wuhan to Guangzhou, by train from Jan. 22 to Jan. 23. She did not neither contact with wild animals, nor eat game meat. On Jan. 24, after lunch, the index case (patient A1) presented with fever and cough at 16:40, and then she presented to the hospital and admitted to the isolation ward with fever, cough and pneumonia feature on CT scans. After the positive result of throat swab by RT-PCR, she was diagnosed as COVID-19 on Jan. 26, all the other 9 family members were classified as close contacts and sent to the Guangzhou No.8 Hospital for isolation quarantine, of which four developed fever symptoms and were diagnosed as COVID-19 cases by RT-PCR from Jan. 27 to Feb. 3.

Patient A1 was the index case. The relationship between the patients was described as below: A2: sister of A1; A3: in-laws of A1, wife of A5; A4: niece of A1; A5: in-laws of A1, husband of A3. B1: wife of B3; B2: daughter of B1 and B3; B3: husband of B1, father of B2. C1: daughter of C2; C2: mother of C1.

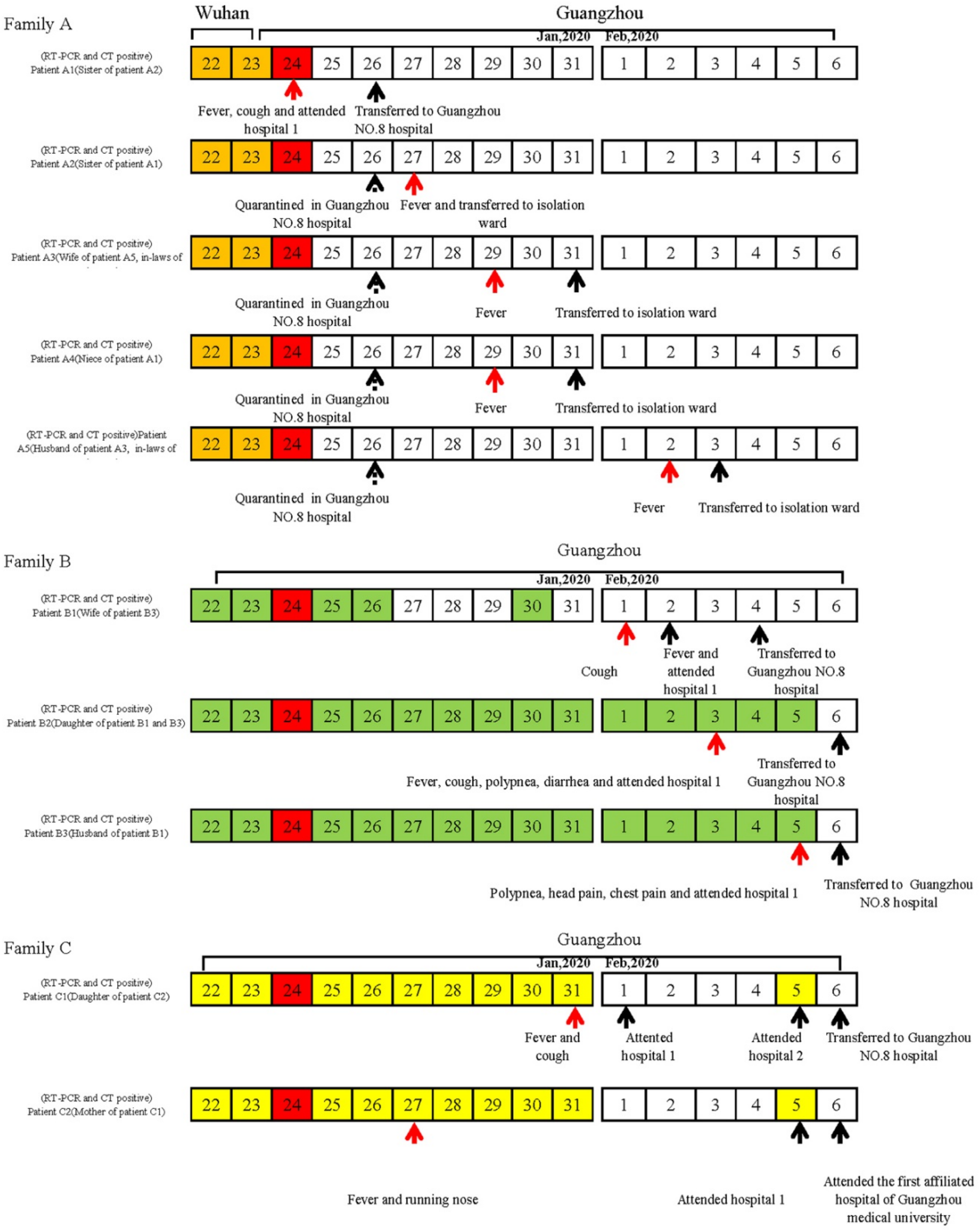

Appendix Figure. Timeline for outbreak of COVID-19 among persons at restaurant, Guangzhou, China, 2020.
